# Insights into the molecular mechanism of allostery in Hsp70s

**DOI:** 10.3389/fmolb.2015.00058

**Published:** 2015-10-20

**Authors:** Matthias P. Mayer, Roman Kityk

**Affiliations:** Zentrum für Molekulare Biologie der Universität Heidelberg (ZMBH), DKFZ/ZMBH Alliance, Ruprecht-Karls-Universität HeidelbergHeidelberg, Germany

**Keywords:** Hsp70 heat-shock proteins, allostery, interdomain communication, conformational dynamics, structure-function relationships

## Abstract

Hsp70s chaperone an amazing number and variety of cellular protein folding processes. Key to their versatility is the recognition of a short degenerate sequence motif, present in practically all polypeptides, and a bidirectional allosteric intramolecular regulation mechanism linking their N-terminal nucleotide binding domain (NBD) and their C-terminal polypeptide substrate binding domain (SBD). Through this interdomain communication ATP binding to the NBD and ATP hydrolysis control the affinity of the SBD for polypeptide substrates and substrate binding to the SBD triggers ATP hydrolysis. Genetic screens for defective variants of Hsp70s and systematic analysis of available structures of the isolated domains revealed some residues involved in allosteric control. Recent elucidation of the crystal structure of the Hsp70 homolog DnaK in the ATP bound open conformation as well as numerous NMR and mutagenesis studies bring us closer to an understanding of the communication between NBD and SBD. In this review we will discuss our current view of the allosteric control mechanism of Hsp70 chaperones.

## Introduction

Hsp70s are involved in a large variety of cellular processes. Thereby they interact with substrate proteins that are in many different conformations: with completely extended polypeptides such as nascent chains at the ribosome (Deuerling and Bukau, 2004; Hartl et al., 2011) or during translocation into organelles (Neupert and Herrmann, 2007; Chacinska et al., 2009); with partially folded and misfolded conformations in late folding intermediates, or upon disaggregation and refolding of stress denatured proteins (Tyedmers et al., 2010); and with native regulatory proteins to control their activity and stability (e.g., heat shock transcription factor σ^32^ in *E. coli* or transcription factors, receptors, and kinases in eukaryotes) (Wegele et al., 2004), and while assisting oligomerization or disassembly of oligomeric structures (e.g., clathrin, Sousa and Lafer, 2015). Hsp70s are ATP dependent chaperones that consist of an N-terminal 45 kDa nucleotide binding domain (NBD) and a 25 kDa substrate polypeptide binding domain (SBD). They do not work alone but interact with cochaperones of the J-domain protein (DnaJ, Hsp40) family, which target Hsp70s to substrate proteins, and several families of nucleotide exchange factors. Hsp70s also cooperate with chaperones of other families like small HSPs and Hsp100s for protein disaggregation, with Hsp90 for regulation of native proteins, with ribosome bound chaperones like trigger factor in prokaryotes and specialized Hsp70s (RAC) in eukaryotes and with Hsp60s for *de novo* folding of proteins. Thus, Hsp70 is probably the most versatile of all chaperones, constituting a central hub of the cellular protein folding network.

One reason for this versatility is most likely the degenerate recognition motif of Hsp70s, which consists of a core of five preferentially hydrophobic amino acids flanked by regions in which positively charged residues are favorable for binding (Rüdiger et al., 1997). Such motifs occur frequently in most proteins. In the folded state these motifs are generally found in the hydrophobic core of the proteins and are exposed only during synthesis when emerging from the ribosomal exit tunnel, during translocation through membranes or during stress denaturation. This explains why Hsp70s interact with most proteins when they are in the denatured but not in the native state. Substrate proteins which interact with Hsp70 in their native conformation apparently expose such sequence motifs even in the completely folded state. Recognition of a short degenerative motif in substrate proteins eliminates any size limitations and Hsp70 can interact with very large proteins and protein complexes, like clathrin cages, or protein aggregates. Another reason for the versatility of the Hsp70 system is certainly the number of J-domain proteins which has increased in the course of evolution from six in *E. coli* and 22 in *S. cerevisiae* to 47 in humans (Kampinga and Craig, 2010). J-domain proteins either bind prospective protein substrates themselves or bind to structures like the ribosomal exit tunnel (e.g., zuotin) or translocation pores (e.g., Sec63, Pam18) where substrates for Hsp70 emerge, and recruit Hsp70 for specific protein folding tasks. Similarly, the different nucleotide exchange factors of three distinct families in the eukaryotic cytosol—the modular multidomain Bag family, the HspBP1 family and the Hsp110 family, which are Hsp70 homologs themselves (Bracher and Verghese, 2015)—may harness Hsp70s for diverse functions.

**Figure 1:**
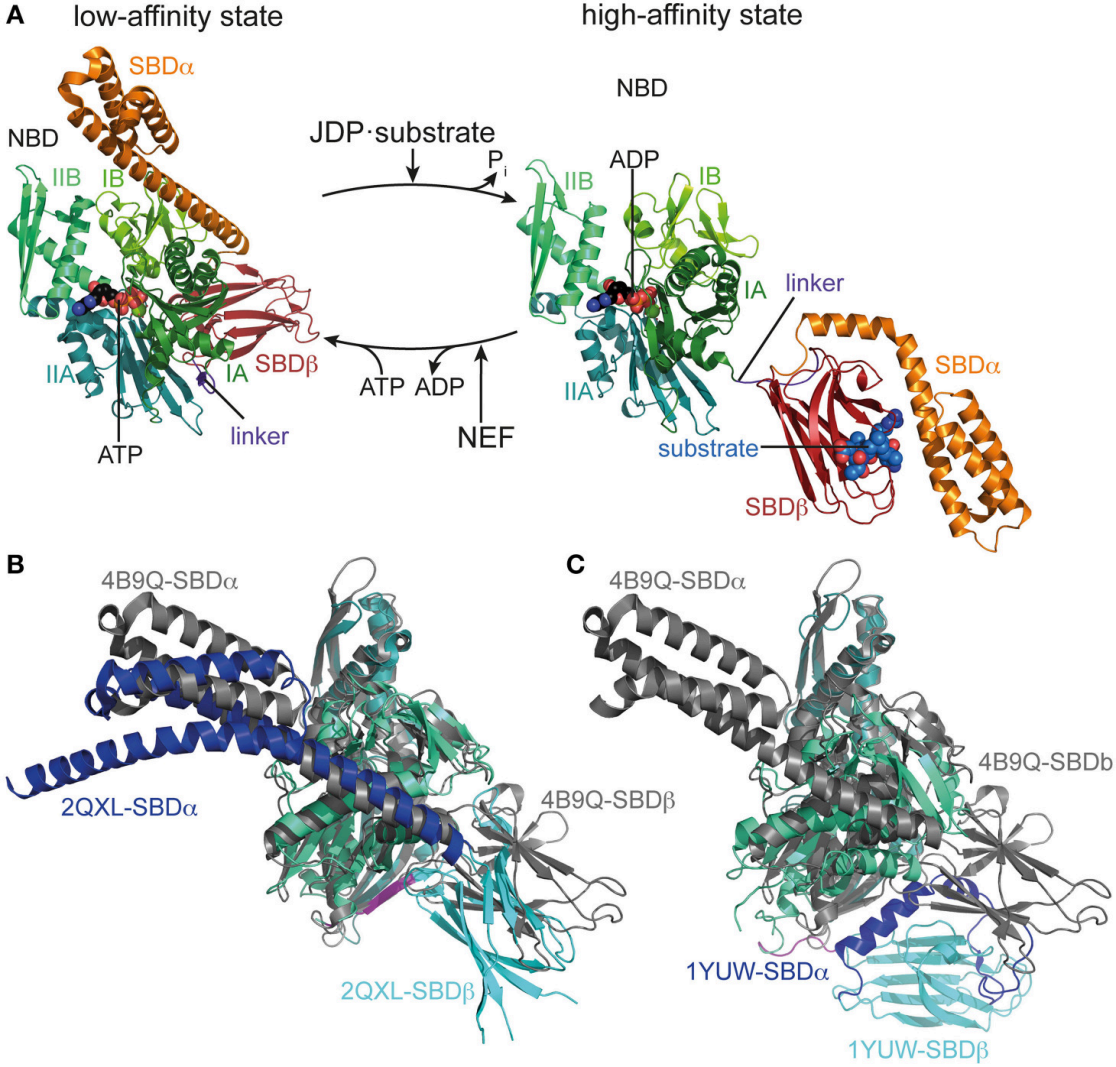
**Conformational cycle of Hsp70s**. **(A)** Structural changes associated with the ATPase cycle of *E. coli* DnaK. Left, crystal structure of DnaK in the ATP bound open conformation (low-affinity state, PDB ID 4B9Q, Kityk et al., 2012) in cartoon representation with NBD subdomains IA, IB, IIA, IIB in different shades of green, SBDβ in dark red and SBDα in orange. Right, solution structure of DnaK in the ADP bound/nucleotide-free state as derived from residual dipolar coupling NMR experiments and crystal structures of the isolated domains (high-affinity state, PDB IDs 2KHO, Bertelsen et al., 2009) colored as in the ATP bound state and NBDs in identical orientation. **(B)** Overlay of the crystal structures of DnaK·ATP (PDB ID 4B9Q; gray) and Sse1 (PDB ID 2QXL, Liu and Hendrickson, 2007); NBD, deep teal; SBDβ, cyan; SBDα, blue. **(C)** Overlay of the crystal structures of DnaK·ATP (PDB ID 4B9Q; gray) and bovine Hsc70(1-554) (PDB ID 1YUW, Jiang et al., 2005) NBD, deep teal; SBDβ, cyan; SBDα, blue.

Finally and most importantly, the intricate mechanism of the Hsp70 machine itself makes it such a versatile tool. In contrast to ATP-independent chaperones, the affinity of Hsp70 for substrates is regulated by nucleotide, the substrate itself, the J-domain cochaperones and nucleotide exchange factors (Figure 1). In a nutshell, in the ATP bound state Hsp70 has a low affinity for substrates but high substrate association and dissociation rates. Upon ATP hydrolysis, substrate association and dissociation rates decrease some 100 and 1000-fold, respectively, leading to an increase in affinity of 10 to 50-fold (Schmid et al., 1994; Mayer et al., 2000). However, ATP hydrolysis rates are very low but stimulated synergistically by the substrate itself and the J-domain cochaperone (Karzai and McMacken, 1996; Barouch et al., 1997; Misselwitz et al., 1998; Laufen et al., 1999; Silberg et al., 2004). Thus, Hsp70 acts like a mouse-trap where the substrate itself triggers its capture. The synergism of substrate with J-domain proteins in triggering ATP hydrolysis allows J-domain proteins to target Hsp70 to the proper substrate. At physiological ATP concentrations, nucleotide exchange is rate-limiting for substrate release and thus allows nucleotide exchange factors to regulate the residence time of substrates bound to Hsp70. This mechanism of association of substrates with Hsp70·ATP at high rates and subsequent ATP hydrolysis and transition to the high affinity state creates a non-equilibrium situation resulting in ultra-high affinity that so far has not been found in any other chaperone (De Los Rios and Barducci, 2014). In the following we will discuss the current knowledge of the structural basis for this allosteric mechanism.

## Structural basis for allostery in Hsp70s

A thorough understanding of the structural basis for allostery in Hsp70s was hampered for many years by the lack of a structure of the full-length protein. Structures of isolated domains (Flaherty et al., 1990; Zhu et al., 1996) were available for many years but information on the assembly of NBD and SBD in ADP or ATP states is rather recent (Jiang et al., 2005; Chang et al., 2008; Bertelsen et al., 2009; Kityk et al., 2012; Qi et al., 2013). The NBD shares structural homology with actin and sugar kinases (Flaherty et al., 1991), and can be divided into two lobes (I and II) with two subdomains each (IA, IB, IIA, IIB), which form a deep cleft at the bottom of which nucleotides bind, contacting all four subdomains (Flaherty et al., 1990, Figure 1). The SBD is composed of a two-layered β-sandwich subdomain (SBDβ), which contains the substrate binding channel with a central pocket capable of binding a single hydrophobic residue of the substrate; an α-helical subdomain (SBDα), consisting of five helices; and a C-terminal intrinsically disordered segment of about 30 residues of unclear function, which seems to be involved in chaperone activity and, in the eukaryotic cytosol, contains the EEVD motif at the very C-terminus, serving as docking site for the cochaperones Hop/Sti1 and Chip (Zhu et al., 1996; Demand et al., 1998; Ballinger et al., 1999; Scheufler et al., 2000; Zhang et al., 2005; Smock et al., 2011). In the isolated SBD, helices A and B of the SBDα are tightly packed onto the SBDβ, enclosing the substrate binding channel like a lid. This also seems to be the most prevalent conformation of the full-length protein in the nucleotide-free and ADP bound states (Jiang et al., 2005; Chang et al., 2008; Bertelsen et al., 2009; Marcinowski et al., 2011; Schlecht et al., 2011). The crystal structure of *Geobacillus kaustophilus* DnaK and NMR studies on *E. coli* DnaK suggest that NBD and SBD are rather separated, independently tumbling units in the nucleotide-free and ADP states only connected by the flexible linker (Swain et al., 2007; Chang et al., 2008; Bertelsen et al., 2009; Zhuravleva et al., 2012). In contrast, the crystal structure of nucleotide-free bovine Hsc70 shows NBD and SBD in a docked conformation (Jiang et al., 2005).

**Figure 2:**
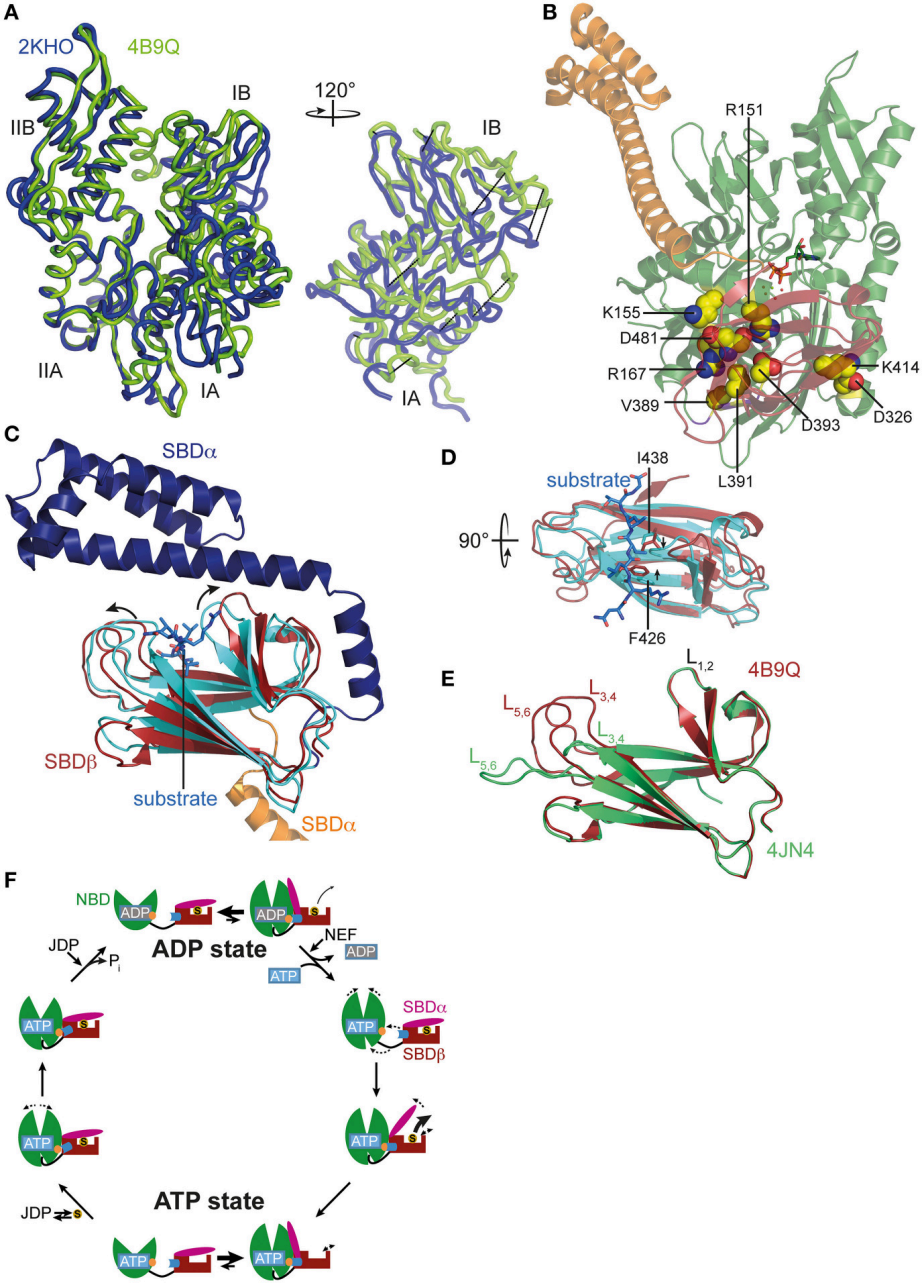
**ATP-induced changes in NBD and SBD and allosteric cycle of Hsp70s**. **(A)** Overlay of the NBD of *E. coli* DnaK in the ATP bound open conformation (PDB ID 4B9Q, Kityk et al., 2012; green) and of DnaK in the nucleotide-free/ADP bound state (PDB ID 2KHO, Bertelsen et al., 2009; blue) in tube representation. Left, standard view; right, only subdomains IA and IB rotated by 120° as indicated. **(B)** Structure of DnaK in the ATP bound open conformation with residues known to be involved in interdomain communication and found in the NBD-SBD interface in space-filling representation with carbon atoms in yellow, oxygen atoms in red and nitrogen atoms in blue. **(C)** Overlay of the SBD of DnaK in the ATP bound open conformation; SBDβ, dark red; SBDα, orange and cut for space reasons and the structure of the isolated SBD in complex with a substrate peptide (PDB ID 1DKX, Zhu et al., 1996); SBDβ, cyan; SBDα, dark blue; peptide in light blue and stick representation. Arrows indicate ATP-induced changes visible in this orientation. **(D)** Overlay of SBDβ as in **(A)**, but rotated by 90° as indicated. Arrows indicate ATP-induced narrowing of the central substrate binding pocket. **(E)** Overlay of the SBDβ of the two available structures of DnaK in the ATP bound open conformation (PDB IDs 4B9Q, Kityk et al., 2012; dark red; 4JN4, Qi et al., 2013; green). Indicated are the substrate enclosing loops *L*_1, 2_, *L*_3, 4_, and *L*_5, 6_. **(F)** In the ADP state Hsp70s are in equilibrium between the closed conformation with NBD (green) and SBD (dark red) only connected via the conserved interdomain linker (black) and substrate (S) tightly enclosed in the substrate binding pocket and a very transient open conformation with NBD and SBDβ (dark red) docked. Since the open conformation is very transient, substrates only dissociate from this state at low rates. Nucleotide exchange factors (NEFs) catalyze ADP dissociation. Subsequent ATP binding to Hsp70 induces rotation of the NBD lobes toward each other, opening of the lower cleft of the NBD, insertion of the conserved interdomain linker, and docking of SBDβ to the NBD, resulting in opening of the α-helical lid (SBDα, magenta) and release of the substrate with high rates. In the ATP state Hsp70s are also in equilibrium between the open and very transient closed conformation. The outer loops of the SBDβ are highly dynamic. Substrates associate with J-domain proteins (JDP) and bind with high rates to the open conformation of Hsp70. Substrate binding induces closing of the SBDα and dissociation of the SBDβ from the NBD, which allows rotation of the NBD lobes to a position optimal for ATP hydrolysis. Substrates stimulate ATP hydrolysis through a distinct pathway (blue) involving a trigger on the NBD (orange). How JDPs act in synergism with substrates is currently not known. Dashed arrows indicate domain movement/dynamics.

Recently, the crystal structure of DnaK in the ATP-bound open conformation was solved, which significantly broadened our knowledge about allostery in Hsp70s (Kityk et al., 2012; Qi et al., 2013). Comparison of the DnaK·ATP structure with the solution structure of DnaK·ADP indicates that ATP binding leads to dramatic structural rearrangements in the protein (Figure 1A). DnaK·ATP has a more compact structure; SBDα and SBDβ are completely detached from each other and docked onto two sides of the NBD; and the interdomain linker is buried in the lower crevice of the NBD. This structure is similar to the structure of the Hsp110 Sse1, which serves as nucleotide exchange factor for Hsp70s (Dragovic et al., 2006; Raviol et al., 2006; Liu and Hendrickson, 2007; Polier et al., 2008; Schuermann et al., 2008) but has clear differences in the structure and orientation of the SBDβ and SBDα (Figure 1B). Differences are more striking when compared to the structure of a two-domain construct of bovine Hsc70, which was crystallized in the nucleotide-free state (Jiang et al., 2005) (Figure 1C). An overlay of the NBD of DnaK·ATP with all previously solved crystal structures of isolated NBDs in complex with different nucleotides and the solution structure of the full-length protein in the ADP state (e.g., Flaherty et al., 1990; Wilbanks et al., 1994; O'Brien et al., 1996; Jiang et al., 2005; Bertelsen et al., 2009) reveals that ATP binding leads to the rotation of the NBD lobes toward each other (Figure 2A and Supplemental Movie 1). This leads to a widening of the lower crevice of the NBD, enabling the linker to insert between subdomains IA and IIA. The surface rearrangements of the NBD allow SBDα and SBDβ docking on the NBD. A number of residues (e.g., Arg151, Arg167, Asp326, Asp393, Lys414, Asp481; all numbers refers to residues in *E. coli DnaK*), which are part of an extensive H-bond network at the NBD-SBDβ interface, were found in genetic and biochemical studies to be important for allosteric signal transmission between the two domains (Montgomery et al., 1999; Vogel et al., 2006a,b; Smock et al., 2010; Kityk et al., 2015) (Figure 2B). Thus, interface stabilization by an H-bond network plays a pivotal role in interdomain communication in Hsp70s. In particular, Asp481, which contacts the NBD subdomain IA, and K414, which contacts NBD subdomain IIA, act like a clamp, fixing the NBD in the ATP bound state and strongly reducing basal ATPase activity in the absence of a trigger provided by substrate binding and interaction with a J-domain protein (Kityk et al., 2015).

ATP-induced docking of the SBD to the NBD leads to the stabilization of the open conformation of the SBD. In SBDα, detached from SBDβ, helices A and B form a continuous helix. The substrate binding cleft in SBDβ is wider as compared to the structure of the isolated DnaK-SBD with bound peptide substrate (PDB code 1DKX; Figures 2C,D), which is consistent with low affinity for polypeptide substrates and high substrate association and dissociation rates in the ATP-state (Schmid et al., 1994; Mayer et al., 2000). The substrate binding cleft is also more open than the NMR structure of the substrate-free isolated SBDβ (PDB code 1DG4) (Pellecchia et al., 2000) This observation suggests that the conformational changes in SBDβ of the DnaK·ATP structure are induced by SBDβ-NBD interactions, and not by the absence of substrate or detachment of SBDα, as was demonstrated recently (Zhuravleva and Gierasch, 2015). Notably, the outer loops of the SBDβ are the only parts which differ significantly between the two crystal structures of DnaK·ATP (PDB codes 4B9Q and 4JN4; Figure 2E) (Kityk et al., 2012; Qi et al., 2013). While in one structure the outer loops *L*_3, 4_ and *L*_5, 6_ protrude upward from the β-sandwich forming a cradle for the substrate (4B9Q), in the other structure they extend the β-strands horizontally (4JN4) (Figure 2E). Although the outer loops seem to be very flexible in the first DnaK·ATP structure as well, as indicated by the high b-factor, it is not clear whether the SBDβ opens to the same extent seen in the second structure. The construct used in the second structure had the outer loop *L*_3, 4_ replaced by a short MGG-motif, and, in the crystal *L*_5, 6_ makes extensive contacts with other molecules that stabilize the extended conformation.

Comparison of the SBDβ of the DnaK·ATP structure with the structure of the isolated SBD with bound substrate (PDB code 1DKX) also suggests a mechanism for how substrates stimulate the ATPase activity. Substrate binding is accompanied by pronounced conformational rearrangements in the SBDβ: (I) the substrate enclosing loop *L*_1, 2_ moves toward the substrate and away from the NBD, (II) binding of the central hydrophobic residue requires an expansion of the substrate binding pocket as compared to the ATP-bound state, leading to an overall reorganization of the SBDβ. These changes are transmitted toward the interface through a defined pathway involving residues Val440, Leu484, and Asp148, presumably leading to disruption of some interdomain contacts and release of the linker from the lower crevice of the NBD (Kityk et al., 2015). As a consequence, the NBD subdomains are able to rotate to a degree where the catalytic residues in the ATPase active center reach the optimal position for γ-phosphate cleavage. Such a model is consistent with NMR measurements on a full-length DnaK construct with bound ATP and substrate peptide, which suggest that substrate binding causes dissociation of the SBDβ from the NBD (Zhuravleva et al., 2012).

Although all of these details on the mechanics of allosteric regulation have been elucidated mainly in *E. coli* DnaK, they are believed to be valid for all Hsp70s due to the high evolutionary conservation of this protein family, albeit, variations of this theme certainly exist, in particular in respect to kinetics of conformational changes.

## Interplay between conformational dynamics and allostery in Hsp70s

Many different studies have demonstrated that Hsp70s are highly dynamic and undergo transitions between open and closed conformations independent of the nucleotide status (Mapa et al., 2010; Marcinowski et al., 2011; Schlecht et al., 2011; Kityk et al., 2012). Thus, each nucleotide state consists of an ensemble of different conformations as originally proposed (Mayer et al., 2000) and nucleotides modulate the frequency of structural transitions and affect the equilibrium between different conformers of Hsp70s. A recent NMR and molecular dynamics study revealed that the SBDβ is much more dynamic in the ATP state than in the ADP state, and that the SBDβ-NBD contacts influence the dynamics of the substrate binding pocket (Zhuravleva and Gierasch, 2015). Based on their data, the authors propose that the substrate binding loops and SBDβ-NBD interface are dynamically coupled and that this coupling is part of the allosteric mechanism.

The conformational equilibrium is also influenced by substrates. Binding of a substrate to Hsp70·ATP seems to induce the closure of the α-helical lid before ATP hydrolysis occurs. Consistent with this notion is the observation that substrate binding reduces the ATP-induced blueshift of Trp102 fluorescence in DnaK, indicating the undocking of the α-helical lid from the NBD even in the absence of ATP hydrolysis (Slepenkov and Witt, 1998; Vogel et al., 2006a). On the other hand, bound substrates were demonstrated to slow ATP-induced docking of the α-helical lid onto the NBD (Kityk et al., 2012). Figure 2F summarizes the current view of allostery and the conformational cycle of Hsp70s.

## Perspectives

Recent years have brought about significant progress in understanding the underlying mechanisms of interdomain communication in Hsp70s. Despite these advances many questions are still not solved. For example, due to the relative scarcity of structural information, the details of the Hsp70-Hsp40 interaction remain elusive. Hence it is not clear how Hsp40s alone, or together with the substrate, influence allosteric signal transmission between the domains. In eukaryotes Hsp70 additionally interacts with other co-chaperones like HOP and CHIP, linking the Hsp70 machinery to the Hsp90 system and the proteasome degradation pathway, respectively. It is not clear whether they alter, either alone or in cooperation with other cochaperones like nucleotide exchange factors, the interdomain communication in Hsp70s to facilitate transfer of the substrate to Hsp90 or to stabilize the Hsp70-substrate complex for timely release at the proteasome. Lastly, it is not known whether interdomain communication is subject to modulation by the post-translational modifications of Hsp70s that occur in eukaryotes (Muller et al., 2012; Truman et al., 2012; Morgner et al., 2015). Interest in the molecular mechanisms of interdomain communication in the Hsp70s is also driven by the prospect of medical applications. Taking into account the important role of Hsp70s in different pathophysiological processes, including cancer, neurodegenerative diseases and autoimmunity, one of the key research areas is development of Hsp70 modulators. The nucleotide binding pocket of Hsp70 was classified as poor inhibitor binding site due to the mostly electrostatic and polar interactions with nucleotide (Halgren, 2009). The polypeptide substrate binding site may be unsui for inhibitors and activators of Hsp70 function and only inhibitors with limited potency have been found so far (Otvos et al., 2000; Otaka et al., 2007; Yamamoto et al., 2010; Knappe et al., 2011). Therefore, allosteric control of the ATPase cycle appears as an attractive target and the first allosteric modulators have already been found (Wisén and Gestwicki, 2008; Kang et al., 2014; Taldone et al., 2014; Hassan et al., 2015).

### Conflict of interest statement

The authors declare that the research was conducted in the absence of any commercial or financial relationships that could be construed as a potential conflict of interest.

## Supplementary material

Supplemental Movie 1 **Lobe rotation in the NBD of Hsp70 upon transition between the ATP and ADP bound state**. The structure of the NBD of *E. coli* DnaK in the ATP bound conformation (PDB ID 4B9Q) was morphed into the structure of the ADP bound conformation (PDB ID 2KHO) using the Yale Morph Server (Krebs and Gerstein, 2000; Flores et al., 2006). Subdomains of the NBD in different shades of green (IA, dark green; IB chartreuse; IIA, dark teal; IIB, lime); NBD-SBD-linker in purple.

## References

[B1] Ballinger C. A. Connell P. Wu Y. Hu Z. Thompson L. J. Yin L. Y.. (1999). Identification of CHIP, a novel tetratricopeptide repeat-containing protein that interacts with heat shock proteins and negatively regulates chaperone functions. Mol. Cell. Biol. 19, 4535–4545. 10330192 10.1128/mcb.19.6.4535 PMC104411

[B2] Barouch W. Prasad K. Greene L. Eisenberg E. (1997). Auxilin-induced interaction of the molecular chaperone Hsc70 with clathrin baskets. Biochemistry 36, 4303–4308. 10.1021/bi962727z 9100026

[B3] Bertelsen E. B. Chang L. Gestwicki J. E. Zuiderweg E. R. P. (2009). Solution conformation of wild-type *E. coli* Hsp70 (DnaK) chaperone complexed with ADP and substrate. Proc. Nat. Acad. Sci. U.S.A. 106, 8471–8476. 10.1073/pnas.0903503106 19439666 PMC2689011

[B4] Bracher A. Verghese J. (2015). The nucleotide exchange factors of Hsp70 molecular chaperones. Front. Mol. Biosci. 2:10 10.3389/fmolb.2015.00010 PMC4753570 26913285

[B5] Chacinska A. Koehler C. M. Milenkovic D. Lithgow T. Pfanner N. (2009). Importing mitochondrial proteins: machineries and mechanisms. Cell 138, 628–644. 10.1016/j.cell.2009.08.005 19703392 PMC4099469

[B6] Chang Y.-W. Sun Y.-J. Wang C. Hsiao C.-D. (2008). Crystal structures of the 70-kDa heat shock proteins in domain disjoining conformation. J. Biol. Chem. 283, 15502–15511. 10.1074/jbc.M708992200 18400763 PMC3258884

[B7] De Los Rios P. Barducci A. (2014). Hsp70 chaperones are non-equilibrium machines that achieve ultra-affinity by energy consumption. Elife 3:e02218. 10.7554/elife.02218 24867638 PMC4030575

[B8] Demand J. Lüders J. Höhfeld J. (1998). The carboxy-terminal domain of Hsc70 provides binding sites for a distinct set of chaperone cofactors. Mol. Cell. Biol. 18, 2023–2028. 9528774 10.1128/mcb.18.4.2023 PMC121432

[B9] Deuerling E. Bukau B. (2004). Chaperone-assisted folding of newly synthesized proteins in the cytosol. Crit. Rev. Biochem. Mol. Biol. 39, 261–277. 10.1080/10409230490892496 15763705

[B10] Dragovic Z. Broadley S. A. Shomura Y. Bracher A. Hartl F. U. (2006). Molecular chaperones of the Hsp110 family act as nucleotide exchange factors of Hsp70s. EMBO J. 25, 2519–2528. 10.1038/sj.emboj.7601138 16688212 PMC1478182

[B11] Flaherty K. M. DeLuca-Flaherty C. McKay D. B. (1990). Three-dimensional structure of the ATPase fragment of a 70K heat-shock cognate protein. Nature 346, 623–628. 10.1038/346623a0 2143562

[B12] Flaherty K. M. McKay D. B. Kabsch W. Holmes K. C. (1991). Similarity of the three-dimensional structures of actin and the ATPase fragment of a 70-kDa heat shock cognate protein. Proc. Natl. Acad. Sci. U.S.A. 88, 5041–5045. 10.1073/pnas.88.11.5041 1828889 PMC51803

[B13] Flores S. Echols N. Milburn D. Hespenheide B. Keating K. Lu J.. (2006). The database of macromolecular motions: new features added at the decade mark. Nucleic Acids Res. 34, D296–D301. 10.1093/nar/gkj046 16381870 PMC1347409

[B14] Halgren T. A. (2009). Identifying and characterizing binding sites and assessing druggability. J. Chem. Inf. Model. 49, 377–389. 10.1021/ci800324m 19434839

[B15] Hartl F. U. Bracher A. Hayer-Hartl M. (2011). Molecular chaperones in protein folding and proteostasis. Nature 475, 324–332. 10.1038/nature10317 21776078

[B16] Hassan A. Q. Kirby C. A. Zhou W. Schuhmann T. Kityk R. Kipp D. R.. (2015). The novolactone natural product disrupts the allosteric regulation of Hsp70. Chem. Biol. 22, 87–97. 10.1016/j.chembiol.2014.11.007 25544045

[B17] Jiang J. Prasad K. Lafer E. M. Sousa R. (2005). Structural basis of interdomain communication in the Hsc70 chaperone. Mol. Cell 20, 513–524. 10.1016/j.molcel.2005.09.028 16307916 PMC4443753

[B18] Kampinga H. H. Craig E. A. (2010). The HSP70 chaperone machinery: J proteins as drivers of functional specificity. Nat. Rev. Mol. Cell Biol. 11, 579–592. 10.1038/nrm2941 20651708 PMC3003299

[B19] Kang Y. Taldone T. Patel H. J. Patel P. D. Rodina A. Gozman A.. (2014). Heat shock protein 70 inhibitors. 1. 2,5′-thiodipyrimidine and 5-(phenylthio)pyrimidine acrylamides as irreversible binders to an allosteric site on heat shock protein 70. J. Med. Chem. 57, 1188–1207. 10.1021/jm401551n 24548207 PMC3983365

[B20] Karzai A. W. McMacken R. (1996). A bipartite signaling mechanism involved in DnaJ-mediated activation of the *Escherichia coli* DnaK protein. J. Biol. Chem. 271, 11236–11246. 10.1074/jbc.271.19.11236 8626673

[B21] Kityk R. Kopp J. Sinning I. Mayer M. P. (2012). Structure and dynamics of the ATP-bound open conformation of Hsp70 chaperones. Mol. Cell 48, 863–874. 10.1016/j.molcel.2012.09.023 23123194

[B22] Kityk R. Vogel M. Schlecht R. Bukau B. Mayer M. P. (2015). Pathways of allosteric regulation in Hsp70 chaperones. Nat. Commun. 6, 8308. 10.1038/ncomms9308 26383706 PMC4595643

[B23] Knappe D. Zahn M. Sauer U. Schiffer G. Sträter N. Hoffmann R. (2011). Rational design of oncocin derivatives with superior protease stabilities and antibacterial activities based on the high-resolution structure of the oncocin-DnaK complex. Chembiochem 12, 874–876. 10.1002/cbic.201000792 21387510

[B24] Krebs W. G. Gerstein M. (2000). The morph server: a standardized system for analyzing and visualizing macromolecular motions in a database framework. Nucleic Acids Res. 28, 1665–1675. 10.1093/nar/28.8.1665 10734184 PMC102811

[B25] Laufen T. Mayer M. P. Beisel C. Klostermeier D. Mogk A. Reinstein J.. (1999). Mechanism of regulation of hsp70 chaperones by DnaJ cochaperones. Proc. Natl. Acad. Sci. U.S.A. 96, 5452–5457. 10.1073/pnas.96.10.5452 10318904 PMC21880

[B26] Liu Q. Hendrickson W. A. (2007). Insights into Hsp70 chaperone activity from a crystal structure of the yeast Hsp110 Sse1. Cell 131, 106–120. 10.1016/j.cell.2007.08.039 17923091 PMC2041797

[B27] Mapa K. Sikor M. Kudryavtsev V. Waegemann K. Kalinin S. Seidel C. A. M.. (2010). The conformational dynamics of the mitochondrial Hsp70 chaperone. Mol. Cell 38, 89–100. 10.1016/j.molcel.2010.03.010 20385092

[B28] Marcinowski M. Höller M. Feige M. J. Baerend D. Lamb D. C. Buchner J. (2011). Substrate discrimination of the chaperone BiP by autonomous and cochaperone-regulated conformational transitions. Nat. Struct. Mol. Biol. 18, 150–158. 10.1038/nsmb.1970 21217698

[B29] Mayer M. P. Schröder H. Rüdiger S. Paal K. Laufen T. Bukau B. (2000). Multistep mechanism of substrate binding determines chaperone activity of Hsp70. Nat. Struct. Biol. 7, 586–593. 10.1038/76819 10876246

[B30] Misselwitz B. Staeck O. Rapoport T. A. (1998). J proteins catalytically activate Hsp70 molecules to trap a wide range of peptide sequences. Mol. Cell 2, 593–603. 10.1016/S1097-2765(00)80158-6 9844632

[B31] Montgomery D. L. Morimoto R. I. Gierasch L. M. (1999). Mutations in the substrate binding domain of the *Escherichia coli* 70 kDa molecular chaperone, DnaK, which alter substrate affinity or interdomain coupling. J. Mol. Biol. 286, 915–932. 10.1006/jmbi.1998.2514 10024459

[B32] Morgner N. Schmidt C. Beilsten-Edmands V. Ebong I.-O. Patel N. A. Clerico E. M.. (2015). Hsp70 forms antiparallel dimers stabilized by post-translational modifications to position clients for transfer to Hsp90. Cell Rep. 11, 759–769. 10.1016/j.celrep.2015.03.063 25921532 PMC4431665

[B33] Muller P. Ruckova E. Halada P. Coates P. J. Hrstka R. Lane D. P.. (2012). C-terminal phosphorylation of Hsp70 and Hsp90 regulates alternate binding to co-chaperones CHIP and HOP to determine cellular protein folding/degradation balances. Oncogene 32, 3101–3110. 10.1038/onc.2012.314 22824801

[B34] Neupert W. Herrmann J. M. (2007). Translocation of proteins into mitochondria. Annu. Rev. Biochem. 76, 723–749. 10.1146/annurev.biochem.76.052705.163409 17263664

[B35] O'Brien M. C. Flaherty K. M. McKay D. B. (1996). Lysine 71 of the chaperone protein Hsc70 Is essential for ATP hydrolysis. J. Biol. Chem. 271, 15874–15878. 10.1074/jbc.271.27.15874 8663302

[B36] Otaka M. Yamamoto S. Ogasawara K. Takaoka Y. Noguchi S. Miyazaki T.. (2007). The induction mechanism of the molecular chaperone HSP70 in the gastric mucosa by Geranylgeranylacetone (HSP-inducer). Biochem. Biophys. Res. Commun. 353, 399–404. 10.1016/j.bbrc.2006.12.031 17182004

[B37] Otvos L. O I. Rogers M. E. Consolvo P. J. Condie B. A. Lovas S.. (2000). Interaction between heat shock proteins and antimicrobial peptides. Biochemistry 39, 14150–14159. 10.1021/bi0012843 11087363

[B38] Pellecchia M. Montgomery D. L. Stevens S. Y. Vander Kooi C. W. Feng H. P. Gierasch L. M.. (2000). Structural insights into substrate binding by the molecular chaperone DnaK. Nat. Struct. Biol. 7, 298–303. 10.1038/74062 10742174

[B39] Polier S. Dragovic Z. Hartl F. U. Bracher A. (2008). Structural basis for the cooperation of Hsp70 and Hsp110 chaperones in protein folding. Cell 133, 1068–1079. 10.1016/j.cell.2008.05.022 18555782

[B40] Qi R. Sarbeng E. B. Liu Q. Le K. Q. Xu X. Xu H.. (2013). Allosteric opening of the polypeptide-binding site when an Hsp70 binds ATP. Nat. Struct. Mol. Biol. 20, 900–907. 10.1038/nsmb.2583 23708608 PMC3772632

[B41] Raviol H. Sadlish H. Rodriguez F. Mayer M. P. Bukau B. (2006). Chaperone network in the yeast cytosol: Hsp110 is revealed as an Hsp70 nucleotide exchange factor. EMBO J. 25, 2510–2518. 10.1038/sj.emboj.7601139 16688211 PMC1478168

[B42] Rüdiger S. Germeroth L. Schneider-Mergener J. Bukau B. (1997). Substrate specificity of the DnaK chaperone determined by screening cellulose-bound peptide libraries. EMBO J. 16, 1501–1507. 10.1093/emboj/16.7.1501 9130695 PMC1169754

[B43] Scheufler C. Brinker A. Bourenkov G. Pegoraro S. Moroder L. Bartunik H.. (2000). Structure of TPR domain-peptide complexes: critical elements in the assembly of the Hsp70-Hsp90 multichaperone machine. Cell 101, 199–210. 10.1016/S0092-8674(00)80830-2 10786835

[B44] Schlecht R. Erbse A. H. Bukau B. Mayer M. P. (2011). Mechanics of Hsp70 chaperones enables differential interaction with client proteins. Nat. Struct. Mol. Biol. 18, 345–351. 10.1038/nsmb.2006 21278757

[B45] Schmid D. Baici A. Gehring H. Christen P. (1994). Kinetics of molecular chaperone action. Science 263, 971–973. 10.1126/science.8310296 8310296

[B46] Schuermann J. P. Jiang J. Cuellar J. Llorca O. Wang L. Gimenez L. E.. (2008). Structure of the Hsp110:Hsc70 nucleotide exchange machine. Mol. Cell 31, 232–243. 10.1016/j.molcel.2008.05.006 18550409 PMC2892728

[B47] Silberg J. J. Tapley T. L. Hoff K. G. Vickery L. E. (2004). Regulation of the HscA ATPase reaction cycle by the co-chaperone HscB and the iron-sulfur cluster assembly protein IscU. J. Biol. Chem. 279, 53924–53931. 10.1074/jbc.M410117200 15485839

[B48] Slepenkov S. V. Witt S. N. (1998). Peptide-induced conformational changes in the molecular chaperone DnaK. Biochemistry 37, 16749–16756. 10.1021/bi981738k 9843445

[B49] Smock R. G. Blackburn M. E. Gierasch L. M. (2011). Conserved, disordered C terminus of DnaK enhances cellular survival upon stress and DnaK *in vitro* chaperone activity. J. Biol. Chem. 286, 31821–31829. 10.1074/jbc.M111.265835 21768118 PMC3173061

[B50] Smock R. G. Rivoire O. Russ W. P. Swain J. F. Leibler S. Ranganathan R.. (2010). An interdomain sector mediating allostery in Hsp70 molecular chaperones. Mol. Syst. Biol. 6, 414. 10.1038/msb.2010.65 20865007 PMC2964120

[B51] Sousa R. Lafer E. M. (2015). The role of molecular chaperones in clathrin mediated vesicular trafficking. Front. Mol. Biosci. 2:26. 10.3389/fmolb.2015.00026 26042225 PMC4436892

[B52] Swain J. F. Dinler G. Sivendran R. Montgomery D. L. Stotz M. Gierasch L. M. (2007). Hsp70 chaperone ligands control domain association via an allosteric mechanism mediated by the interdomain linker. Mol. Cell 26, 27–39. 10.1016/j.molcel.2007.02.020 17434124 PMC1894942

[B53] Taldone T. Kang Y. Patel H. J. Patel M. R. Patel P. D. Rodina A.. (2014). Heat shock protein 70 inhibitors. 2. 2,5′-thiodipyrimidines, 5-(phenylthio)pyrimidines, 2-(pyridin-3-ylthio)pyrimidines, and 3-(phenylthio)pyridines as reversible binders to an allosteric site on heat shock protein 70. J. Med. Chem. 57, 1208–1224. 10.1021/jm401552y 24548239 PMC3983364

[B54] Truman A. W. Kristjansdottir K. Wolfgeher D. Hasin N. Polier S. Zhang H.. (2012). CDK-dependent Hsp70 phosphorylation controls G1 cyclin abundance and cell-cycle progression. Cell 151, 1308–1318. 10.1016/j.cell.2012.10.051 23217712 PMC3778871

[B55] Tyedmers J. Mogk A. Bukau B. (2010). Cellular strategies for controlling protein aggregation. Nat. Rev. Mol. Cell Biol. 11, 777–788. 10.1038/nrm2993 20944667

[B56] Vogel M. Bukau B. Mayer M. P. (2006a). Allosteric regulation of Hsp70 chaperones by a proline switch. Mol. Cell 21, 359–367. 10.1016/j.molcel.2005.12.017 16455491

[B57] Vogel M. Mayer M. P. Bukau B. (2006b). Allosteric regulation of Hsp70 chaperones involves a conserved interdomain linker. J. Biol. Chem. 281, 38705–38711. 10.1074/jbc.M609020200 17052976

[B58] Wegele H. Müller L. Buchner J. (2004). Hsp70 and Hsp90–a relay team for protein folding. Rev. Physiol. Biochem. Pharmacol. 151, 1–44. 10.1007/s10254-003-0021-1 14740253

[B59] Wilbanks S. M. DeLuca-Flaherty C. McKay D. B. (1994). Structural basis of the 70-kilodalton heat shock cognate protein ATP hydrolytic activity. I. kinetic analyses of active site mutants. J. Biol. Chem. 269, 12893–12898. 8175706

[B60] Wisén S. Gestwicki J. E. (2008). Identification of small molecules that modify the protein folding activity of heat shock protein 70. Anal. Biochem. 374, 371–377. 10.1016/j.ab.2007.12.009 18191466

[B61] Yamamoto S. Nakano S. Owari K. Fuziwara K. Ogawa N. Otaka M.. (2010). Gentamicin inhibits HSP70-assisted protein folding by interfering with substrate recognition. FEBS Lett. 584, 645–651. 10.1016/j.febslet.2009.12.021 20026329

[B62] Zhang M. Windheim M. Roe S. M. Peggie M. Cohen P. Prodromou C.. (2005). Chaperoned ubiquitylation–crystal structures of the CHIP U box E3 ubiquitin ligase and a CHIP-Ubc13-Uev1a complex. Mol. Cell 20, 525–538. 10.1016/j.molcel.2005.09.023 16307917

[B63] Zhu X. Zhao X. Burkholder W. F. Gragerov A. Ogata C. M. Gottesman M. E.. (1996). Structural analysis of substrate binding by the molecular chaperone DnaK. Science 272, 1606–1614. 10.1126/science.272.5268.1606 8658133 PMC5629921

[B64] Zhuravleva A. Clerico E. M. Gierasch L. M. (2012). An interdomain energetic tug-of-war creates the allosterically active state in Hsp70 molecular chaperones. Cell 151, 1296–1307. 10.1016/j.cell.2012.11.002 23217711 PMC3521165

[B65] Zhuravleva A. Gierasch L. M. (2015). Substrate-binding domain conformational dynamics mediate Hsp70 allostery. Proc. Nat. Acad. Sci. 112, E2865–E2873. 10.1073/pnas.1506692112 26038563 PMC4460500

